# Oil Adsorption Kinetics of Calcium Stearate-Coated Kapok Fibers

**DOI:** 10.3390/polym15020452

**Published:** 2023-01-15

**Authors:** Aimee Lorraine M. Blaquera, Marvin U. Herrera, Ronniel D. Manalo, Monet Concepcion Maguyon-Detras, Cybelle Concepcion M. Futalan, Mary Donnabelle L. Balela

**Affiliations:** 1 Sustainable Electronic Materials Group, Department of Mining, Metallurgical, and Materials Engineering, University of the Philippines Diliman, Quezon City 1101, Metro Manila, Philippines; 2 Institute of Mathematical Sciences and Physics, College of Arts and Sciences, University of the Philippines Los Baños, Los Baños 4031, Laguna, Philippines; 3 Department of Forest Products and Paper Science, College of Forestry and Natural Resource, University of the Philippines Los Baños, Los Baños 4031, Laguna, Philippines; 4 Department of Chemical Engineering, College of Engineering and Agro-Industrial Technology, University of the Philippines Los Baños, Los Baños 4031, Laguna, Philippines; 5 Department of Community and Environmental Resource Planning, College of Human Ecology, University of the Philippines Los Baños, Los Baños 4031, Laguna, Philippines

**Keywords:** kapok fiber, calcium stearate, oil sorption, adsorption kinetics

## Abstract

This study used a simple and efficient dipping method to prepare oleophilic calcium stearate-coated kapok fibers (CaSt_2_-KF) with improved hydrophobicity. Fourier transform infrared spectroscopy (FTIR), X-ray diffraction (XRD), and scanning electron microscopy (SEM) confirmed the deposition of calcium stearate particles on the surface of the kapok fibers. This led to higher surface roughness and improved static water contact angle of 137.4°. The calcium stearate-coated kapok fibers exhibited comparable sorption capacities for kerosene, diesel, and palm oil. However, the highest sorption capacity of 59.69 g/g was observed for motor oil at static conditions. For motor oil in water, the coated fibers exhibited fast initial sorption and a 65% removal efficiency after 30 s. At equilibrium, CaSt_2_-KF attained a sorption capacity of 33.9 g/g and 92.5% removal efficiency for motor oil in water. The sorption kinetics of pure motor oil and motor oil in water follows the pseudo-second-order kinetic model, and the Elovich model further described chemisorption. Intraparticle diffusion and liquid film diffusion were both present, with the latter being the predominant diffusion mechanism during motor oil sorption.

## 1. Introduction

Oil remains as one of the most valuable resources in this rapidly evolving human life having various domestic, recreational, and industrial applications. Catering to the need for oil entails a growing demand for storage and transport [1]. These activities have led to accidental and intentional oil releases in large bodies of water, resulting in billions of tons of discharged oil [2]. As extensive negative environmental and economic impacts persist from the effects of oil pollution, immediate mitigation to lessen the damages is deemed necessary [2,3]. Oil spill remediation techniques include chemical [4], direct combustion [5], bioremediation [6], and physical remediation methods [7]. The most appropriate approach is selected based on several factors such as location, quantity, type of spilled oil, etc. Aside from these factors, the consequences of inducing secondary pollution should be considered. These challenges continue the call for efficient and cost-effective technologies to recover oil in polluted waters [7,8].

Sorbents have been proven to have excellent oil removal efficiency and manageability. Inorganic sorbents, such as perlite, fly ash, exfoliated graphite, and other synthetic sorbents, experience major drawbacks in terms of eco-friendliness, reusability, and cost [9,10]. The search for sustainable sorbent materials led to the development of natural sources and waste products due to their abundance and biodegradability [11,12], which addresses high production costs and lack of waste disposal strategies for spent sorbents [13]. Cellulose-based sorbents were reported to have similar or even better oil sorption performance than synthetic sorbents [14,15]. Cellulosic fibers, such as poplar seed [16], nettle [17], water hyacinth [18], rice straw [19], cotton [20], and kapok fibers [21], have been studied for their oil sorption capabilities. However, cellulosic materials also exhibit high water wettability, limiting their application in oil-in-water sorption [22].

Kapok fiber is an emerging oil sorbent with inherent hydrophobic-oleophilic properties. The hollow lumen structure and fiber assembly create substantial spaces for oil uptake [23,24]. Although the fibers are predominantly cellulosic, their waxy surface renders them low wettability to water. Additionally, kapok fibers’ low density and excellent buoyancy make them suitable for recovering spilled oil [25,26,27]. Surface modifications are made to improve oil adhesion and retention by increasing surface roughness or coating with an oleophilic material. However, retaining the hollow lumen structure of kapok and improving its hydrophobicity during modification remains a challenge for its development as an oil sorbent [25]. Additionally, reported modifications of kapok fibers often utilize expensive precursor materials, such as silanes and metal oxides [28,29,30], or complicated methods involving high temperatures and freeze-drying [31,32,33].

The sensitivity of spilled oil to chemical and physical changes over time necessitates prompt oil recovery. Although the degradation process of oil is usually slow, the unpredictable marine environment conditions, such as waves, currents, temperature, adds to the difficulty in handling such pollutants [14,34]. Therefore, the development of alternative sorbent materials should consider the importance of evaluating the sorption behavior and mechanisms during oil recovery [35]. The sorption kinetics and mechanisms of various oils by raw and treated kapok fibers were previously investigated [36,37]. 

In this study, calcium stearate was coated on the surface of the kapok fiber via an efficient one-step dipping method done at room temperature to improve the surface roughness and oil retention of the kapok fibers. Pure oil and oil-in-water sorption by calcium stearate-coated kapok fibers (CaSt_2_-KF) were performed. The hydrophobicity, sorption performance, and reusability of the kapok fibers with various commercial oils were investigated. The applicability of the experimental data to different kinetic models was evaluated to understand the sorption behavior and kinetic parameters of CaSt_2_-KF. Through the modification of kapok fibers and evaluation of their sorption performance and behavior, this study aimed to provide a better understanding on their suitability for pure oil and oil-in-water applications.

## 2. Materials and Methods

### 2.1. Materials

Kapok fibers were obtained from local kapok trees in Los Baños, Laguna, Philippines. They were separated from their hulls and seeds and cleaned with distilled water and technical grade ethanol (CH_3_CH_2_OH, 95%, RTC). Calcium chloride dihydrate (CaCl_2_•2H_2_O, Techno Pharmchem), stearic acid (C_18_H_36_O_2_, RTC), analytical grade ethanol (CH_3_CH_2_OH, 100% undenatured, Chemsupply), and sodium hydroxide (J.T. Baker) were used as received. Commercially available kerosene (SEAOIL), diesel (Petron), palm oil (UFC Golden Fiesta), and motor oil (Shell Helix HX3 SAE-40 monograde motor oil) were used for oil sorption experiments. The viscosity of the commercial oils from Table 1 was obtained using ASTM D1545-76 at 25 °C.

### 2.2. Preparation of CaSt_2_-Coated Kapok Fibers

Raw kapok fibers (raw-KF) were cleaned and dried at 70 °C for 3 h. The calcium stearate (CaSt_2_) solution was prepared by mixing 40 mL of 0.6 M CaCl_2_ solution and 20 mL of 0.6 M stearic acid in ethanol solution. With constant stirring at 650 rpm, 20 mL of 0.12 M NaOH solution was added dropwise. Afterward, 0.8 g of raw-KF was immersed in the CaSt_2_ solution for 2 min and dried at 80 °C for 6 h. The immersion and drying process was repeated to ensure the coating of CaSt_2_. 

### 2.3. Material Characterizations

Scanning electron microscopy (SEM, JEOL JIB-4000 Plus) was performed to evaluate the surface morphology of CaSt_2_-KF. Surface area analysis was done using Brunauer–Emmett–Teller Method (BET, Quantachrome Instruments NOVA 2200e Surface Area and Pore Analyzer). The chemical composition and surface functional groups were determined using X-ray diffraction analysis (XRD, Shimadzu XRD-6100), energy dispersive X-ray analysis (EDX, JEOL JIB-4000 Pl), and Fourier transform infrared spectroscopy (FTIR, ThermoScientific Nicolet iS50). Static water contact angles were obtained using a digital microscope (Dino-Lite AM2111-0.3MP USB). Measurements were performed three times in randomized areas at room temperature with ~10 µL water droplet volume. 

### 2.4. Oil Sorption and Reusability Experiments

Raw-KF and CaSt_2_-KF were weighed and immersed in 25 mL of motor oil. After 30 min, the fibers were removed, drained to remove residual oil, and then weighed. Reusability tests were performed by subsequent squeezing and immersing in model oil multiple times. The oil sorption capacity (*q*) was calculated using Equation (1):

(1) $$q=\frac{m_{f}-m_{i}}{m_{i}}$$

where *m_f_* is the weight of the oil-ridden fiber and *m_i_* is the weight of the dry fiber. According to previous studies, tests beyond 30 min are not feasible since sorption of petroleum products usually achieves saturation before this time, and actual oil spill events also require fast removal durations to prevent oil desorption and degradation of sorbent [38,39,40].

### 2.5. Sorption Kinetic Studies

#### 2.5.1. Pure Oil Sorption

The pure oil sorption of CaSt_2_-KF was performed based on literature [41]. In a 150 mL beaker, 0.1 g of CaSt_2_-KF was immersed in 100 mL of motor oil. The fibers were retrieved at specific time intervals (10 s–30 min), drained for 30 s without squeezing, and then weighed. The process was repeated until the equilibrium or maximum sorption capacity was obtained. The sorption capacities at each time interval (*q_t_*) were calculated using Equation (1). 

#### 2.5.2. Oil-In-Water Sorption

Oil-in-water sorption of CaSt_2_-KF was performed based on literature [42]. The oil-in-water mixture was prepared by placing 4 mL of motor oil and 100 mL distilled water in a 150 mL beaker at a minimal agitation rate of 200 rpm. 0.1 g of CaSt_2_-KF was placed on the oil-in-water mixture for specific amounts of time (0.5–10 min), drained for 30 s, and then weighed. The fibers were dried at 105 °C for 24 h to remove sorbed water, and the oil-ridden fibers were reweighed. The sorption capacities at each time interval (*q_t_*) were calculated using Equation (1). The removal efficiency (*RE*) was also calculated using Equation (2):

(2) $$RE=\frac{m_{f}-m_{i}}{m_{i,oil}}\times 100\%$$

where *m_i,oil_* is the weight of motor oil in the oil-in-water mixture. 

#### 2.5.3. Kinetic Models

Various kinetic models were used in determining the sorption behavior involved in oil-only and oil-in-water sorption. Table 2 shows the linear and nonlinear equations of the kinetic models used to fit the experimental data from the sorption of motor oil by CaSt_2_-KF. Model fitting and parameter calculations were conducted using Microsoft Excel. Correlation coefficient (R^2^) and chi-square square analysis (𝜒^2^) were used to determine the suitability of the linear and nonlinear fitted models for the system, respectively. 

The main sorption mechanisms were examined using the pseudo-first-order, pseudo-second order, and Elovich kinetic models. Suitability to the pseudo-first-order model indicates a reversible system wherein physical sorption is the primary sorption mechanism. Linear fitting involves plotting ln(*q_e_ − q_t_*) vs. *t*, where *q_e_* (g/g) and *q_t_* (g/g) are the sorption capacities at equilibrium and specific time intervals, respectively, *t* is the immersion time, and *k*_1_ is the pseudo-first-order rate constant [39,43]. Meanwhile, the pseudo-second order model indicates both physical and chemisorption in the system. Linear fitting involves plotting *t/q_t_* vs. *t*, obtaining the pseudo-second-order rate constant *k*_2_ [39,42]. Additionally, the initial sorption rate *h* can be calculated using Equation (3):

(3) $$h=k_{2}q_{e}^{2}$$

Elovich kinetic model is used to evaluate the initial sorption rate 𝛼 and rate constant 𝛽 which describes the activation energy and extent of chemisorption [41,43]. 

Diffusion mechanisms were further evaluated using intraparticle diffusion and liquid film diffusion models. Intraparticle diffusion refers to the transport of oil from the liquid phase towards both pore and surface diffusion. Pore diffusion is expected to be rate-limiting when the intercept *I* or boundary layer effect is zero upon plotting *q_t_* vs. *t*^1/2^. A higher intercept means having surface diffusion as rate-limiting in the sorption process. The intraparticle diffusion rate constant *k_d_* may also be obtained [41,44]. To further evaluate surface sorption, liquid film diffusion modeling was performed. Liquid film diffusion refers to the transport of oil from the liquid bulk towards the film surrounding the surface of the fibers. Linear plotting of ln(*1 − F*) vs. *t*, where *F* is the fractional attainment of equilibrium (*F = q_t_/q_e_*), generates the liquid film diffusion rate constant *k_fd_*. A zero intercept suggests that liquid film diffusion would be rate-limiting [44,45].

## 3. Results and Discussion

### 3.1. Modification of Kapok Fibers with Calcium Stearate

**Figure 1:**
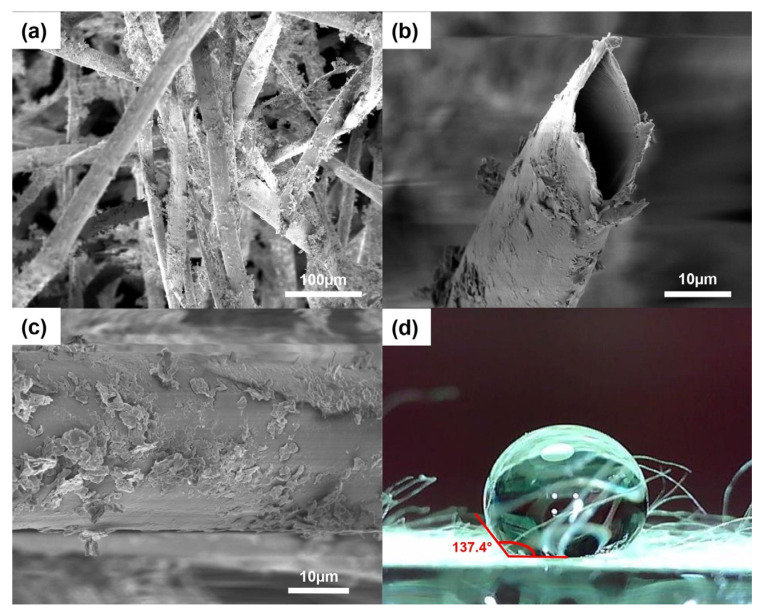
(Color online) SEM images of (**a**) CaSt_2_-KF at low magnification (300×), (**b**) its cross-section and (**c**) surface at high magnification (2000×), and (**d**) CaSt_2_-KF’s static water contact angle measurement.

Figure 1 shows the SEM images and water contact angle measurement of the modified kapok fibers. The hollow tubular structure was maintained after coating with CaSt_2_, as seen in Figure 1a,b. The cross-sectional image in Figure 1b shows an oval-shaped lumen and a thin fiber wall. The fibers exhibited an inner diameter in the range of 20–22 µm, which is consistent with the previously reported values from raw-KF [26]. Figure 1c shows randomly dispersed plate-like calcium stearate particles embedded on the fiber surface. The coating appears to be non-uniformly distributed, considering the presence of dense aggregations of CaSt_2_ and bare fiber surfaces. Nevertheless, the addition of CaSt_2_ particles provided surface roughness on the previously smooth waxy layer of raw-KF. The unaffected hollow lumen structure of the fibers and the added surface roughness from the CaSt_2_ particles provide effective space for oil sorption in the fiber assembly. The increase in surface roughness increases the surface area that anchors oil particles on the fiber surface during sorption and improves oil retention, especially during the extraction of the sorbent from the remediated media [25,46,47]. Surface area analysis using BET showed that modification increased the surface area of raw-KF (0.355 m^2^/g). CaSt_2_-KF exhibited a surface area of 7.024 m^2^/g. 

**Figure 2:**
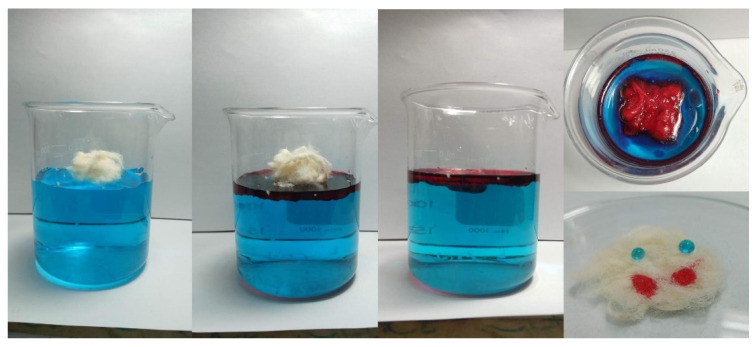
(Color online) Surface wettability test of CaSt_2_-KF on water (dyed with methyl blue) and palm oil (dyed with oil red).

The coating also rendered the fibers more hydrophobic with a static water contact angle of 137.4°, as seen from Figure 1d. This value improves 129° for raw-KF obtained from a previous study [27]. The hydrophobicity of CaSt_2_-KF is proven further by a simple surface wettability test as seen in Figure 2, which shows the high selectivity of the fibers to oil as it readily adsorbed onto the surface of the fibers upon contact. The CaSt_2_-KF also maintained its buoyancy despite being ridden with oil on the water surface, which is essential in oil spill remediation. 

**Figure 3:**
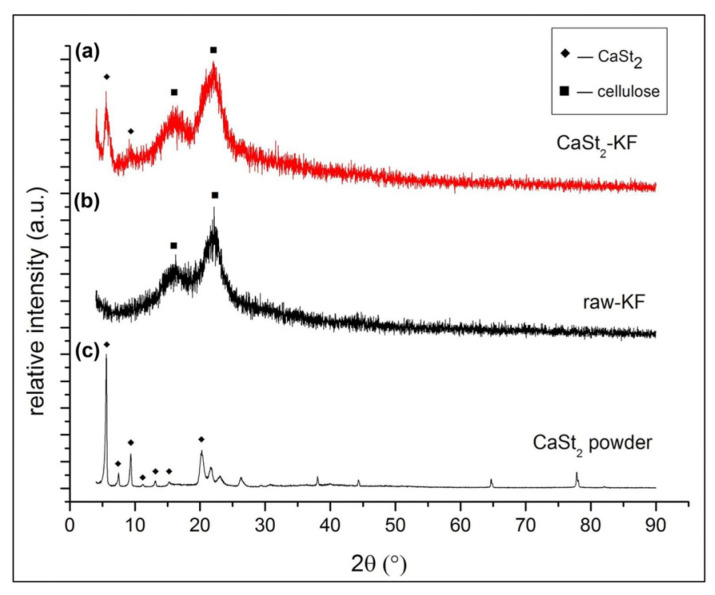
(Color online) XRD patterns of (**a**) CaSt_2_-KF, (**b**) raw-KF, (**c**) synthesized CaSt_2_ powder.

Figure 3 shows the XRD patterns of CaSt_2_-KF, raw-KF, and CaSt_2_-KF powders. The diffraction peaks at 2𝜃 = 5.58, 9.10, 15.60, and 22.33° characteristics of CaSt_2_-KF are seen in Figure 3a. The diffraction peaks at 2𝜃 = 15.60 and 22.33° refer to the crystalline components of cellulose [48]. These are the only characteristic peaks from raw-KF, as seen in Figure 3b. Cellulose in kapok fibers may be amorphous and crystalline. The amorphous cellulose components in kapok fibers are found primarily on the outer wall of the fibers, while crystalline components are in the inner wall [48,49]. The presence of CaSt_2_ on kapok fibers is indicated by the diffraction peaks at 2𝜃 = 5.58 and 9.10°, which were observed from the XRD pattern of synthesized CaSt_2_ powders from Figure 3c. CaSt_2_ powders were prepared with the same process of CaSt_2_-KF sans the kapok fibers. The diffraction peaks at 2𝜃 = 5.63, 7.48, 9.36, 11.18, 13.18, an 15.28° shown in Figure 3c represent the crystalline bilayers of CaSt_2_ [50]. The broadening of some peaks in the spectra of CaSt_2_-KF in Figure 3a may have been influenced by the amorphous components of kapok fibers and the presence of non-uniform coating [48].

**Figure 4:**
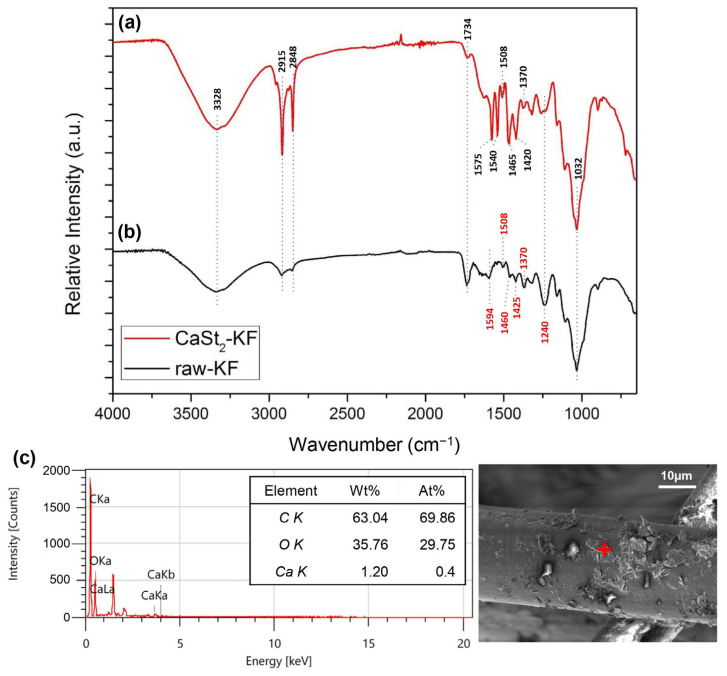
(Color online) FTIR spectra of (**a**) CaSt_2_-KF and (**b**) raw-KF, and (**c**) EDX spectra of CaSt_2_-KF (‘+’ is the point of analysis).

**Figure 5:**
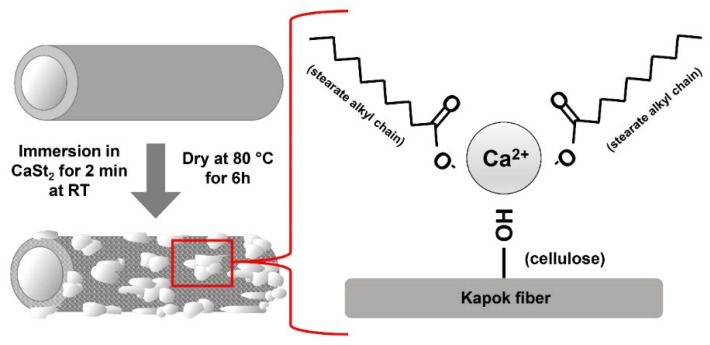
(Color online) Schematic representation of the one-step modification of kapok fibers with calcium stearate.

The FTIR spectra of CaSt_2_-KF and raw-KF can be seen in Figure 4a and Figure 4b, respectively. The presence of the broad –OH– stretching vibration peak at 3328 cm^−1^ and C–O stretching vibration peak at 1032 cm^−1^ in both spectra represents the waxy cutin and cellulosic components of the kapok fibers [26,51,52]. The peaks at 2915 cm^−1^ and 2848 cm^−1^ belong to the asymmetric and symmetric –CH– stretching vibrations, which may be associated with the cellulose and plant wax [25,53]. The spectra of CaSt_2_-KF in Figure 4a showed an increase in intensity for the peaks at 2915 cm^−1^ and 2848 cm^−1^, indicating the presence of CaSt_2_ [52]. Plant wax is also characterized by the peaks at 1734 cm^−1^ and 1370 cm^−1^ corresponding to C=O stretching vibrations and C=O acetyl group stretching at 1240 cm^−1^. These peaks correspond to the aliphatic aldehydes, esters, and ketones of plant wax [53,54]. CaSt_2_-KF showed a decrease in intensity for these peaks, which indicates the possible deesterification of kapok fibers due to the presence of NaOH in the CaSt_2_ solution [47]. The skeletal C=C stretching vibrations at 1594 cm^−1^, 1504 cm^−1^, and 1460 cm^−1^ of lignin and –CH– stretching vibrations at 1425 cm^−1^ are all present in raw-KF. However, only the peak at 1504 cm^−1^ remained for CaSt_2_-KF. The other peaks were masked by the addition of new pronounced COO– asymmetric stretching vibration peaks at 1575 cm^−1^ and 1540 cm^−1^ and COO– symmetric stretching vibration peaks at 1465 cm^−1^ and 1420 cm^−1^ corresponding to CaSt_2_ [50,52]. The decrease in the intensity of peaks attributed to plant wax and lignin in the spectra of CaSt_2_-KF may have exposed the cellulosic structure of the kapok fibers as indicated by the strong and broad –OH group peak. This –OH group has the potential to chemically bond with the CaSt_2_ coating [55]. As shown in the schematic in Figure 5, CaSt_2_ is composed of a Ca^2+^ ion head and two long alkyl chain tails. An ionic or dipole-dipole interaction between the positive Ca^2+^ of the coating and negative –OH of the cellulose exposed on the fiber surface may have occurred during the modification of the kapok fibers. The presence of CaSt_2_ on the kapok fiber surface is further confirmed by the appearance of Ca element through the EDX analysis from Figure 4c. The low amount of Ca may be due to the irregular coating of CaSt_2,_ as seen from the SEM images. Nevertheless, the surface analysis showed that calcium stearate successfully anchored onto the surface of the kapok fibers.

### 3.2. Oil Sorption Capacity

**Figure 6:**
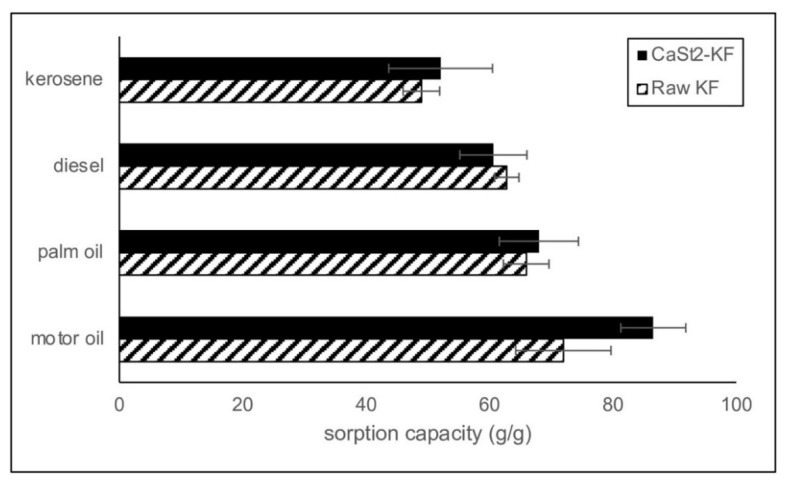
Comparison of the sorption capacities of CaSt_2_-KF and raw-KF in various model oils.

Various oils were utilized to evaluate the sorption performance of the modified kapok fibers. The maximum sorption capacities of CaSt_2_-KF in different oils are higher than those of raw-KF in Figure 6. CaSt_2_-KF showed a maximum sorption capacity of 52.07, 60.63, 68.00, and 86.55 g/g in kerosene, diesel, palm oil, and motor oil, respectively. Oil viscosity significantly influenced the sorption performance of the modified and raw kapok fibers. CaSt_2_-KF exhibited the highest sorption capacity for motor oil, which has the highest viscosity, as listed in Table 1. Low viscosity oils like kerosene and diesel rapidly form a film around the smooth surface of the raw kapok fibers and penetrate the internal lumen. However, low viscous oils can also easily escape or desorb [16]. High viscosity oils like palm oil and motor oil are more likely to be anchored onto the surface. Penetration is still possible for high viscosity oils, but the rate would be slower [37]. In this case, the highly viscous oil is entrapped within the fiber assembly through the effect of stronger capillary bridging. The additional surface roughness provided by calcium stearate particles rendered the surface with greater adhesion to highly viscous oils [8,26,56].

### 3.3. Reusability

**Figure 7:**
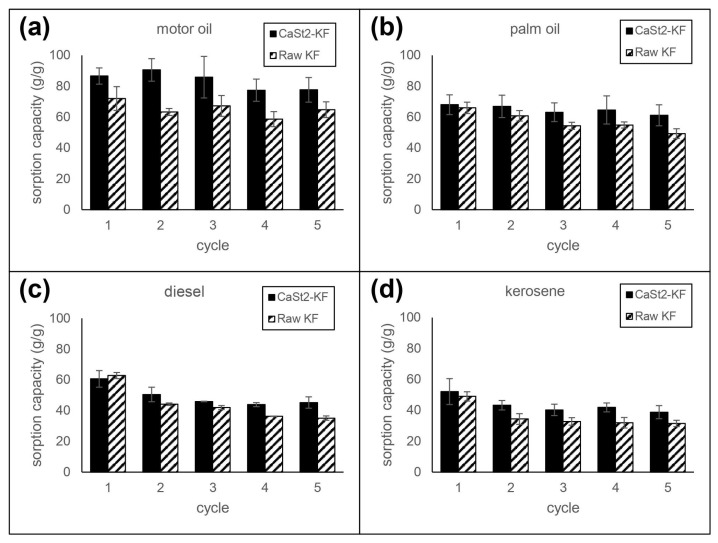
Reusability of CaSt_2_-KF and raw-KF in (**a**) motor oil, (**b**) palm oil, (**c**) diesel, and (**d**) kerosene.

The reusability of CaSt_2_-KF and raw-KF sorbents was investigated by multiple cycles of immersion and squeezing in various model oils. The sorption capacity per cycle is referenced from the initial mass of the fibers. Reusability test results can be seen in Figure 7. CaSt_2_-KF attained sorption capacities of 38.70, 45.23, 61.12, and 77.61 g/g after five sorption cycles for kerosene, diesel, palm oil, and motor oil, respectively, at 30 min each cycle. These values are higher than that of raw-KF. Consequent squeezing may result in deformation of the hollow lumen, increased compaction of the fiber assembly, and trapped residual oil inside the fibers. The decrease in available spaces for oil uptake decreases the sorption capacity over multiple cycles [26,47]. High percent retention (>89%) for highly viscous oils was observed for CaSt_2_-KF and raw-KF. However, a decrease in retention was seen for low viscous oils, where CaSt_2_-KF exhibited ≥75% retention after five sorption cycles. The improved surface roughness in CaSt_2_-KF may have hindered these oils from easily escaping the fiber assembly compared to raw-KF with a smooth surface [16,57].

### 3.4. Kinetic Modeling

#### 3.4.1. Pure Oil Sorption

**Figure 8:**
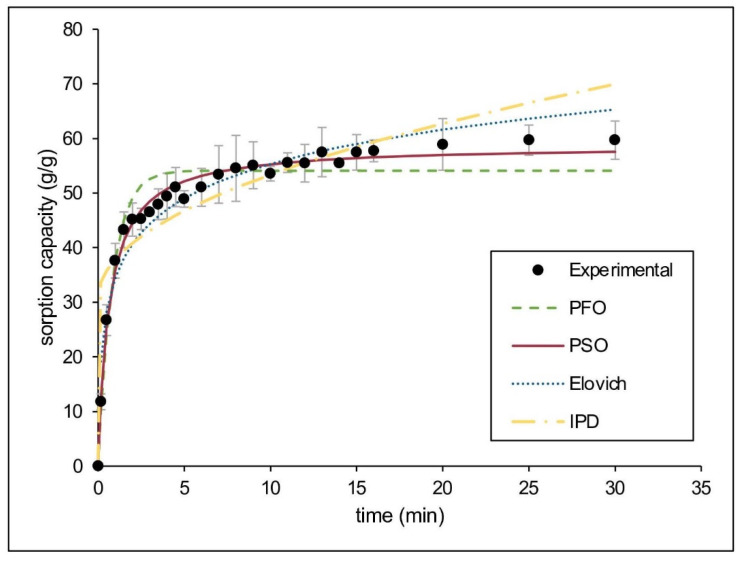
(Color online) Kinetic modeling of experimental data for motor oil sorption by CaSt_2_-KF.

Considering the excellent performance of CaSt_2_-KF for sorption of motor oil in static conditions, kinetic modeling was done to estimate the sorption mechanism. Figure 8 shows the experimental data obtained and the nonlinear fitted models. The kinetic parameters and applicability indicators for both linear and nonlinear fitting of models are presented in Table 3. The system fitted best for the pseudo-second-order kinetic model having the highest R^2^ (0.9989), lowest 𝜒^2^ (1.1979) values, and closest *q_e_*, 60.6061 g/g for linear and 58.7719 g/g for nonlinear, to the experimental *q_e_* (59.6887 g/g). The agreement to this model suggests that both physical and chemisorption are present during oil sorption. The Elovich kinetic model further describes the heterogeneous nature of pseudo-second-order kinetics [58]. The system exhibited high R^2^ (0.9318) and low 𝜒^2^ (6.3266) values for this model, suggesting that chemisorption may be the rate limiting step [41,59].

The diffusion mechanisms were also estimated using intraparticle diffusion and liquid film diffusion models. The mass transfer mechanism varies during different stages of oil sorption, and diffusion models determine which stage is rate-limiting. The stages of sorption include (1) external mass transfer from liquid bulk to fiber surface; (2) liquid film diffusion; and (3) intraparticle diffusion [41,60]. The external mass transfer is rapid, as seen in Figure 8, which is therefore not rate-limiting. Upon modeling, both intraparticle diffusion and liquid film diffusion models do not pass through the origin. Neither can be assumed as the sole rate-limiting step [61]. The intraparticle diffusion model has shown a non-zero intercept (*I*), indicating the presence of a boundary layer effect. The large *I* indicates that surface diffusion is rate-limiting compared to pore diffusion. Liquid film diffusion exhibited a higher R^2^ (0.9236) than the intraparticle diffusion model (R^2^ = 0.7139), suggesting that surface sorption may be the predominant diffusion mechanism for the system. Surface sorption is advantageous in reusability since desorption requires minimal energy [44].

#### 3.4.2. Oil-In-Water Sorption

**Figure 9:**
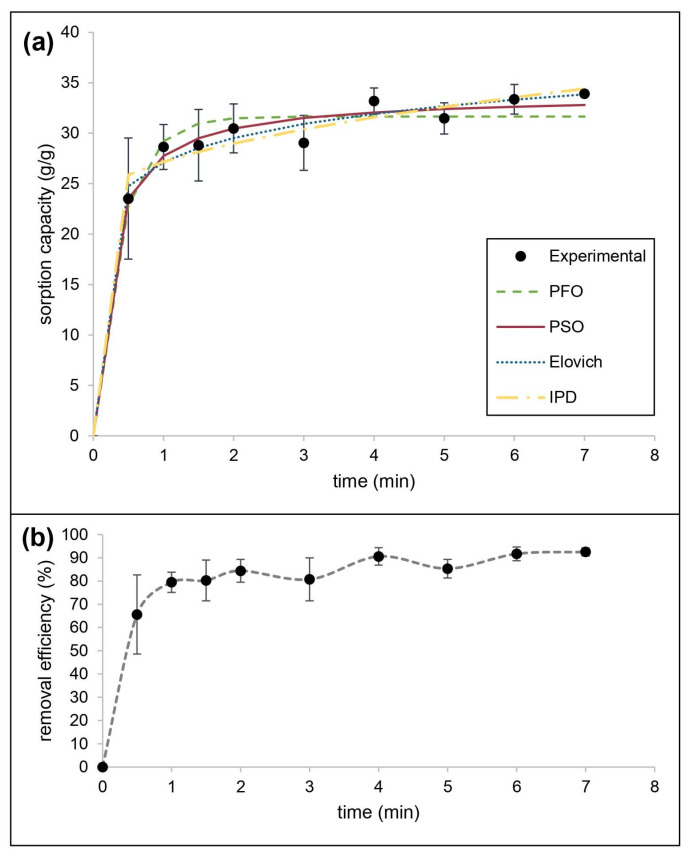
(Color online) (**a**) Kinetic modeling of experimental data and (**b**) removal efficiency for motor oil sorption by CaSt_2_-KF.

The mechanism of sorption of motor oil mixed with water by CaSt_2_-KF was also estimated using kinetic models. Figure 9 shows the experimental data obtained, the nonlinear fitted models, and the motor oil removal efficiency by CaSt_2_-KF. The kinetic parameters and applicability indicators for both linear and nonlinear fitting of models were presented in Table 4. The system consistently fitted best for the pseudo-second-order kinetic model showing the highest R^2^ (0.9959) and lowest 𝜒^2^ (0.3625) values. It also produced the closest *q_e_*, 34.7222 g/g for linear and 33.8150 g/g for nonlinear, compared to the experimental *q_e_* (33.9245 g/g). This is explained by the rapid initial sorption by CaSt_2_-KF, exhibiting a 65.61% removal efficiency after only 30 s [62]. At equilibrium, 92.5% removal efficiency was reached. The system also showed a good fit (R^2^ = 0.8659) with the Elovich model, indicating the presence of chemisorption.

The rapid initial sorption is governed by external mass transfer diffusion. Meanwhile, both the intraparticle diffusion model and liquid diffusion showed low linearity having a non-zero intercept (I), suggesting that both contribute to the rate-limiting step. Intraparticle diffusion exhibited a higher R^2^ (0.8073) than the liquid film diffusion model (R^2^ = 0.7344). Given high magnitudes of *I* from intraparticle diffusion modeling, surface sorption is still identified as the predominant diffusion mechanism for the system [44].

## 4. Conclusions

The potential use of calcium stearate-coated kapok fibers to remove oil in oil-in-water applications was evaluated. As shown by XRD, FTIR, and SEM-EDX analyses, calcium stearate was successfully coated on the kapok fibers. The modified kapok fibers showed improved hydrophobicity with a water contact angle of 137.4° owing to the roughened fiber surface by calcium stearate particles. CaSt_2_-KF had similar sorption performance for kerosene, diesel, and palm oil and improved sorption capacity for motor oil compared to raw-KF. Greater sorption capacities were observed for high viscosity oils. Consequently, CaSt_2_-KF exhibited ≥ 75% retention after five cycles for all model oils, with improved retention for low viscous oils. Kinetic experiments showed that motor oil only and oil-in-water sorption both agreed to pseudo-second-order kinetic model. Chemisorption was also present with high suitability to the Elovich model. Surface diffusion was the predominant diffusion mechanism for both systems. The improved surface properties and sorption performance for pure oil and oil-in-water systems make CaSt_2_-KF a suitable sorbent material, especially for oil-in-water applications.

## Figures and Tables

**Table 1:** Viscosity of model oils at room temperature.

Model Oil	Viscosity (cSt)
kerosene	<54
diesel	<54
palm oil	54–69
motor oil	302–335

**Table 2:** Summary of linear and nonlinear equations of kinetic models and their parameters.

Kinetic Model	Linear Form	Nonlinear Form	Parameters
Pseudo-First Order (PFO)	$\ln\left(q_{e}-q_{t}\right)=\ln\left(q_{e}\right)-k_{1}\left(t\right)$	$q_{t}=q_{e}\left(1-e^{-k_{1}t}\right)$	*k*_1_ [min^−1^] *q_e_* [g g^−1^]
Pseudo-Second Order (PSO)	$\frac{t}{q_{t}}=\frac{1}{k_{2}q_{e}^{2}}+\frac{t}{q_{e}}$	$q_{t}=\frac{q_{e}^{2}k_{2}t}{q_{e}k_{2}t+1}$	*k*_2_ [g g^−1^ min^−1^] *q_e_* [g g^−1^] *h* [g g^−1^ min^−1^]
Elovich	$q_{t}=\frac{\ln\left(\alpha \beta \right)}{\beta }+\frac{\ln\left(t\right)}{\beta }$	$q_{t}=\frac{1}{\beta }\left(\ln\left(\alpha \beta t+1\right)\right)$	𝛼 [g g^−1^ min^−1^] 𝛽 [g g^−1^]
Intraparticle Diffusion (IPD)	$q_{t}=k_{d}t^{1/2}+I$	$q_{t}=k_{d}t^{1/2}+I$	*k_d_* [g g^−1^ min^−1/2^] *I* [g g^−1^]
Liquid Film Diffusion (LFD)	$\ln\left(1-F\right)=-k_{fd}t$	N/A	*k_fd_* [min^−1^]

**Table 3:** Summary of kinetic model parameters from linear and nonlinear curve fitting of experimental data for motor oil sorption by CaSt_2_-KF.

Kinetic Models	Linear		Nonlinear	
PFO	R^2^	0.9236	𝜒^2^	6.5209
	*k*_1_	0.1641	*k_1_*	1.1979
	*q_e_*	24.3785	*q_e_*	54.0882
PSO	R^2^	0.9987	𝜒^2^	1.1399
	*k*_2_	0.0186	*k_2_*	0.0268
	*q_e_*	60.6061	*q_e_*	58.7719
	*h*	68.4932	*h*	92.4950
Elovich	R^2^	0.9318	𝜒^2^	6.3266
	𝛼	501.3912	𝛼	393.3614
	𝛽	0.1152	𝛽	0.1097
IPD	R^2^	0.7139	𝜒^2^	22.7360
	*k_d_*	7.5790	*k_d_*	7.1616
	*I*	29.5970	*I*	30.7190
LFD	R^2^	0.9236	N/A*	
	*k_fd_*	0.1641		

* Nonlinear curve-fitting is not applicable to the liquid film diffusion model.

**Table 4:** Summary of kinetic model parameters from linear and nonlinear curve fitting of experimental data for motor oil-in-water sorption by CaSt_2_-KF.

Kinetic Models	Linear		Nonlinear	
PSO	R^2^	0.7344	𝜒^2^	0.7585
	*k*_1_	0.4410	*k*_1_	2.5566
	*q_e_*	10.2420	*q_e_*	31.6637
PSO	R^2^	0.9959	𝜒^2^	0.3625
	*k*_2_	0.0932	*k*_2_	0.1350
	*q_e_*	34.7222	*q_e_*	33.8150
	*h*	112.3596	*h*	154.3231
Elovich	R^2^	0.8659	𝜒^2^	0.3880
	𝛼	9685.2331	𝛼	8977.4790
	𝛽	0.2929	𝛽	0.2898
IPD	R^2^	0.8073	𝜒^2^	0.5762
	*k_d_*	4.3519	*k_d_*	4.4024
	*I*	22.8190	I	22.7643
LFD	R^2^	0.7344	N/A*	
	*k_fd_*	0.4410		

* Nonlinear curve-fitting is not applicable to the liquid film diffusion model.

## Data Availability

The data presented in this study are available on request from the corresponding author.
